# The blocking effect of the glycoprotein IIb/IIIa receptor in the mouse model of asthma

**DOI:** 10.1186/s12948-021-00149-6

**Published:** 2021-07-13

**Authors:** Seo-Hee Kim, Hoang Kim Tu Trinh, Hae-Sim Park, Yoo Seob Shin

**Affiliations:** 1 Department of Biomedical Science, University School of Medicine, Suwon, South Korea; 2 Center for Molecular Biomedicine, University of Medicine and Pharmacy, Ho Chi Minh City, Vietnam; 3 Department of Allergy and Clinical Immunology, Ajou University School of Medicine, Worldcup-ro 164, Yeoungtong-gu, 443-380 Suwon-si, South Korea

**Keywords:** Asthma, Eosinophil, Glycoprotein, Platelet

## Abstract

**Background:**

It is apparent that the interaction between platelets and eosinophils plays a critical role in the activation of allergic inflammation. We investigated whether blocking of the glycoprotein (GP) IIb/IIIa receptor can attenuate allergic inflammation and airway hyperresponsiveness through inhibition of platelet–eosinophil aggregation (PEA) in asthma.

**Methods:**

BALB/c mice were sensitized by intraperitoneal injection of ovalbumin (OVA) on days 0 and 14, followed by 3 nebulized OVA challenges on days 28–30. On each challenge day, 5 mg/kg tirofiban was administered intraperitoneally 30 min before the challenge. Mice were assessed for airway hyperresponsiveness (AHR), airway inflammation, and the degree of PEA. Finally, the activation levels of platelets and eosinophils were evaluated.

**Results:**

Tirofiban treatment decreased AHR and eosinophilic inflammation in Bronchoalveolar Lavage (BAL) fluid. This treatment also reduced the levels of interleukin (IL)-4, IL-5, and IL-13 in BAL fluid and airway inflammatory cell infiltration in histological evaluation. Interestingly, the blocking of the GP IIb/IIIa receptor more reduced PEA in both blood and lung tissue of tirofiban-treated mice than in those of the positive control mice, and both eosinophilic and platelet activations were attenuated in tirofiban-treated mice.

**Conclusions:**

The blocking of GP IIb/IIIa receptor with tirofiban can attenuate AHR and airway inflammation through the inhibition of PEA and activation.

## Introduction

Platelets are well-known primary cells in the hemostasis and tissue repair process, and they are also known as effector cells in chronic inflammatory diseases such as asthma [1]. Platelet activation has been observed in allergic diseases since 1972 [2], and increased release of chemokines and mediators from platelets, such as platelet factor (PF) 4 and RANTES, were found in asthmatic patients after bronchial challenge [3, 4]. Asthmatic patients have also been reported to have mild thrombocytopenia [5] and to have more shortened platelet lifespan in the circulation than healthy controls [6]. These results mean that continuous platelet activation may play a role in the pathogenesis of asthma.

The platelet–leukocyte interaction is a fundamental cellular process that is characterized by the exchange of signals between platelets and different types of leukocytes in atherosclerosis and inflammatory reactions. This interaction can amplify the synthesis of pro-inflammatory compounds such as the leukotrienes and thromboxane A2. In asthma, the frequency of platelet-adherent leukocytes, especially eosinophils, is strikingly increased in the blood of aspirin-exacerbated respiratory disease (AERD) patients [7], and the degree of platelet–leukocyte aggregation (PEA) are elevated in the blood and nasal polyp of AERD where cysteinyl leukotrienes are their main pathogenesis [8]. Moreover, AERD patients who had greater baseline platelet activation completely inhibited all aspirin-induced symptoms after treatment with P2Y12 receptor antagonism, prasugrel [9]. Platelet–leukocyte interactions are also found in other inflammatory diseases except asthma. For example, platelet activation and coagulation abnormalities were found in inflammatory bowel disease patients [10], and the platelet to lymphocyte ratio was correlated with the disease severity of systemic lupus erythematosus patients [11]. These results highlight the potential for the role of platelets in the pathogenesis of inflammatory diseases [12]. However, the clinical significance of platelet activation in pathologic mechanisms and its use for asthma therapy have not been elucidated. Asthma is a chronic inflammatory disease of the respiratory tract, and many asthmatics are eosinophil-dominant. Therefore, most asthmatic patients are well controlled with inhaled corticosteroid treatment [13]. However, a considerable number of asthmatic patients are not controlled after treatment with an inhaled corticosteroid, which leads to an urgent unmet need for new therapeutic strategies.

When platelets are activated and aggregated with leukocytes, they can directly interact with other cells via contact-dependent or soluble mediator-dependent mechanisms [14]. P-selectin/P-selectin glycoprotein ligand 1 (PSGL-1), CD40/CD40 ligand, and the glycoprotein (GP) IIb/IIIa receptor are involved in contact-dependent interactions [7], and cysteinyl leukotrienes, which can bind to P2Y12 receptor, are well known as soluble mediator-dependent factors. In our previous study, the inhibition of the P2Y12 receptor, which is a soluble mediator-dependent factor in clopidogrel treatment, has been shown to attenuate airway inflammation and airway hyperresponsiveness through diminishing the platelet–eosinophil interaction as well as platelet‐dependent eosinophil recruitment and degranulation in the asthma mouse model [15]. However, there have been few studies on direct contact-dependent interactions between platelets and leukocytes in asthma. The GP IIb/IIIa receptor is known as a major factor for contact-dependent interactions between platelets and leukocytes [16], and the blocking effect of the GP IIb/IIIa receptor by tirofiban which is a GP IIb/IIIa receptor inhibitor widely used for cardiovascular and antithrombotic effects [17].

In this study, we hypothesized that blocking of the direct contact-dependent interaction between platelets and eosinophils will reduce the formation of PEA as well as platelet and eosinophil activations. We also compared the blocking effects of GP IIb/IIIa and P2Y12 in an eosinophilic asthma model, which could decrease asthma symptoms in an eosinophilic asthma model and have the therapeutic potential for new asthma treatment.

## Materials and methods

### Animals

Female BALB/c mice (6 weeks old; weight, 20 ± 2 g) were obtained from the Jackson Laboratory (Bar Harbor, ME, USA). The animals were housed under specific pathogen-free conditions and were maintained on a 12-h light/dark cycle with food and water provided *ad libitum*. All animal experiments conducted in this study were approved by the Institutional Animal Care and Use Committee of Ajou University (IACUC 2017-0068).

### Eosinophilic asthma mouse model and tirofiban treatment

BALB/c mice were intraperitoneally sensitized with 10 µg/mg ovalbumin (OVA) and aluminum hydroxide solution on days 0 and 14, followed by 3 nebulized OVA challenges on days 28–30 using an ultrasonic nebulizer (NE-SM1; Ktmed Inc., Seoul, South Korea) [18]. On each challenge day, 5 mg/kg tirofiban was administered intraperitoneally 30 min before the challenge. The mice were assayed for the next experiments 48 h after the last challenge.

### Airway resistance measurement and sample collection

The FlexiVent system (Scireq, Montreal, QC, Canada) was employed to measure airway resistance. On the day indicated, the mice were anesthetized with pentobarbital sodium and intubated with a cannula. After connecting them to a computer-controlled small-animal ventilator, the mice were ventilated with a tidal volume of 10 mL/kg at a frequency of 150 breaths/min. The baseline airway resistance (R_L_) of each mouse was recorded. Subsequently, a dilution series of acetyl-β-methylcholine chloride (MCh) from 3.12 to 50 mg/mL were gradually introduced to the mice, and the R_L_ values at each concentration were recorded.

### Harvest of bronchoalveolar lavage (BAL) fluid and lung histology analysis

After measuring airway resistance, BAL fluid was harvested with a wash of 1 mL of PBS plus 2 % bovine serum albumin (Sigma Aldrich, St. Louis, MO, USA). After the BAL fluid was centrifuged at 1500 rpm for 5 min at 4 °C, leukocytes were quantified with a hemocytometer, and differential cell counts were performed by counting at least 200 cells on cytospin slides stained with Wright–Giemsa stain. The supernatant was collected and stored at − 70 °C until further analysis. Tissue sections were evaluated using ImageJ (National Institutes of Health, Bethesda, MD, USA). To detect inflammatory cells, sections were stained with haematoxylin and eosin, and mucus-containing cells were stained with periodic acid-Schiff (PAS). The number of inflammatory cells per µm^2^ of perivascular and peribronchial areas and the number of mucus-containing cells per µm^2^ of basement membrane were determined.

### Measurement of cytokine levels

The levels of IL-4, IL-5, IL-13 (eBioscience, San Diego, CA, USA), PF-4 (Abcam, Cambridge, UK), and eosinophilic cationic protein (ECP) (MyBiosource, Inc., San Diego, CA, USA) in the BAL fluid were measured by enzyme-linked immunosorbent assay (ELISA) according to the manufacturer’s instructions.

### Identification of PEA in whole blood

Flow cytometry of PEA in whole blood was performed as previously described [19]. Mouse whole blood was harvested by cardiac puncture into tubes containing 3.8 % sodium citrate to prevent coagulation. The whole blood was incubated with phycoerythrin (PE)-conjugated anti-mouse Siglec-F and fluorescein isothiocyanate (FITC)-conjugated anti-mouse CD41 for 30 min at room temperature (RT) in the dark. Next, red blood cells (RBCs) were lysed with the RBC Lysis/Fixation solution (Biolegend, San Diego, CA, USA) for 10 min and washed once with 1× PBS. The cells were analyzed immediately by flow cytometry with the BD FACSCanto II (BD Bioscience, San Diego, CA, USA) as previously described [19]. A leukocyte gate was set in terms of size and granularity. Within the leukocyte gating, eosinophils were labeled by PE-conjugated anti-mouse Siglec-F. PEA was identified as CD41^+^ eosinophils (Siglec-F^+^/CD41^+^), and at least 1000 events were recorded for each sample. A pooled sample from 3 mice was used to obtain a sufficient number of eosinophils for the analysis.

### Immunofluorescence (IF)

IF was performed using the immunofluorescence technique on 5 μm-thick paraffin sections. After deparaffinization, tissue sections were sequentially incubated with blocking buffer (0.05 % PBS-Tween 20 containing 5 % bovine serum albumin and 10 % normal donkey serum) for 1 h at RT and then incubated with rabbit anti-eosinophil peroxidase (EPX) antibody (Bioss Antibodies), rat anti-PSGL-1 antibody (Ray Biotech) overnight at 4 °C. For IF labeling, the sections were incubated with appropriate secondary antibodies. The Alexa Fluor 594 donkey anti-rat antibody (Life Technologies) and Alexa Fluor 488 donkey anti-rabbit antibody (Life Technologies) were applied for 50 min at 37 °C. Finally, after nuclear staining with DAPI (0.5 µg/mL) for 5 min, biomeda mounting solution (Biomeda) was dropped on the glass slide, and the coverslips were inverted and placed onto glass microscope slides.

### Detection MAC-1 of by western blotting

Thirty-five micrograms of protein was isolated from each tissue homogenate with RIPA buffer. Proteins was separated 12 % SDS-PAGE gel and transferred onto polyvinylidene difluoride (PVDF) membranes (Bio-Rad, Hercules, CA, USA). Blocking in 5 % skim milk (Sigma Aldrich) in Tris buffered saline containing 0.01 % Tween 20 (TBST-T) for 1 h at room temperature. The membranes were probed with primary antibodies against MAC-1 (bs-1014R, Bioss antibodies) and β-actin antibody (sc-47,778, Santa Cruz Biotechnology) was used as an internal control. After extensive washing in Tween-TBS, The membranes were incubated with biotinylated secondary antibody for 1 h at room temperature. Antibody binding was visualized using an ECL detection kit (GE Healthcare, Little Chalfont, UK), and images were acquired using a gel doc system (Bio-Rad Laboratories, Inc., Hercules, CA, USA).

### Antibodies and reagents

The antibodies used against the mouse target proteins were anti-PSGL-1 antibody (Q14242; Ray Biotech, USA), anti-EPX antibody (bs-3881R; Bioss Antibodis), Alexa Fluor 488-conjugated donkey anti-rabbit IgG (A21206), and Alexa Fluor 594-conjugated donkey anti-rat IgG (A21209) (ThermoFisher Scientific, Waltham, MA, USA). Peridinin chlorophyll protein complex (PerCP)-conjugated anti-Ly6G (127,654, Biolegend), allophycocyanin (APC)-conjugated CD11c (117,309, Biolegend), PE-conjugated anti-Siglec-F (552,126, BD Bioscience), FITC-conjugated anti-CD41 (133,904, Biolegend), and PE/Cy7-conjugated anti-CD62P (148,310, Biolegend) antibodies were used for flow cytometry and cell sorting. All drugs used for treatment were obtained from Sigma-Aldrich unless indicated otherwise.

### Statistical analysis

Data are presented as mean ± standard error. The differences between treatment groups were assessed using one-way analysis of variance and Tukey’s post hoc test unless indicated otherwise. The differences between the stimulated and control cells in the in vitro assay were assessed using the Wilcoxon signed-rank test. All statistical analyses were performed by using SPSS software version 23.0 (SPSS Inc., Chicago, IL, USA), and a *P*-value of < 0.05 was considered significant.

## Results

### Tirofiban administration attenuates AHR and airway inflammation in BAL fluid and lung tissue

**Fig. 1:**
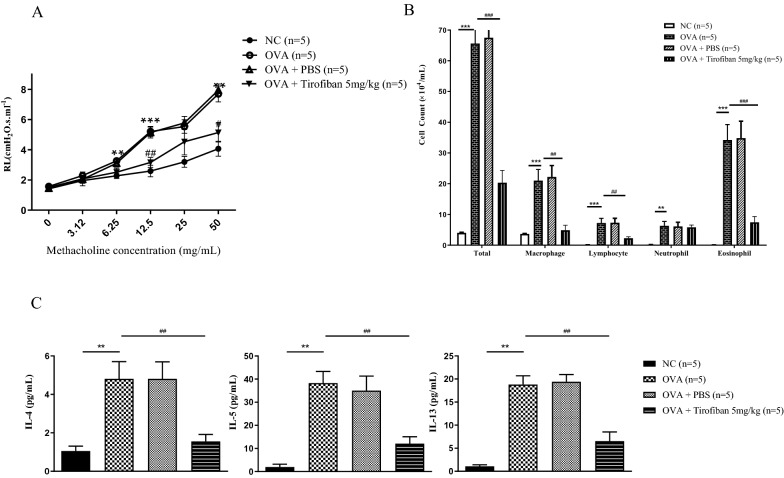
The effects of tirofiban in airway hyper‐responsiveness and inflammation. *P < 0.05, **P < 0.01, ***P < 0.001 between the NC and OVA groups. #P < 0.05, ##P < 0.01 between the OVA and OVA/Tirofiban groups

To determine the therapeutic effects of tirofiban on OVA-induced AHR and airway inflammation in the eosinophilic asthma model, mice were treated with 5 mg/kg tirofiban at the OVA-challenge phase. Airway responses to inhaled MCh were more attenuated and eosinophil counts were more decreased in tirofiban-treated mice than in the positive control mice *(P < 0.05)* (Fig. 1A, B). Also, the BAL fluid levels of IL-4, IL-5, and IL-13 were more reduced in the tirofiban-treated mice than in the positive control mice *(P < 0.01)* (Fig. 1).

**Fig. 2:**
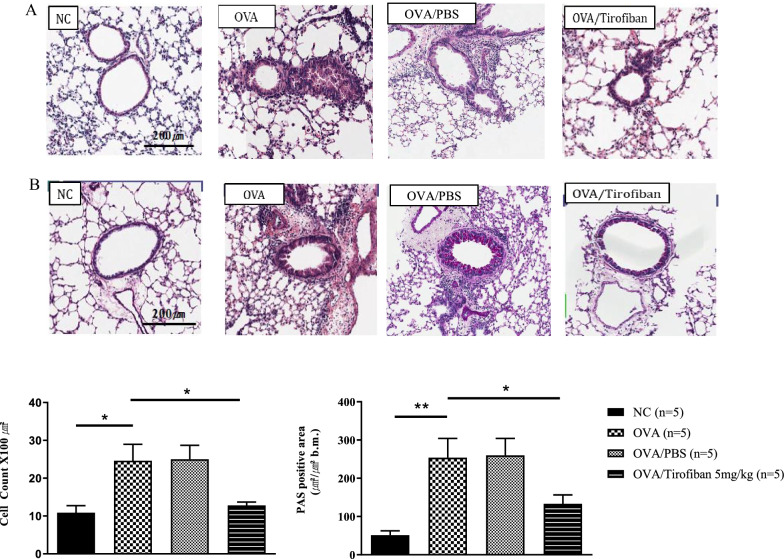
The effects of tirofiban in lung tissue. Lung tissues were stained with H&E stain (**A) **and with PAS stain (**B**). *P < 0.05, **P  < 0.01 between the groups indicated

Histopathological examination revealed peribronchial and perivascular inflammatory cell infiltration in the positive control mice; however, tirofiban treatment significantly decreased the numbers of inflammatory cells and PAS-positive mucus-containing goblet cells in lung tissue (*P < 0.05*) (Fig. 2).

### Tirofiban suppresses PEA in mouse blood

**Fig. 3:**
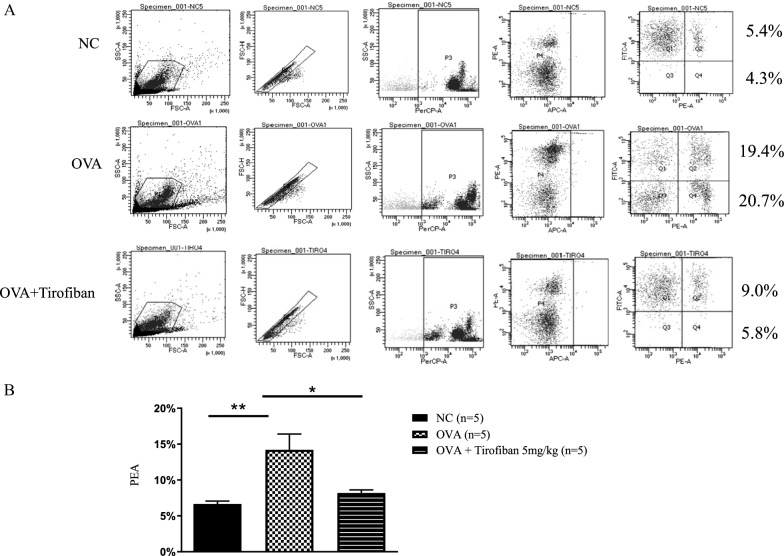
PEA in blood. Flow cytometry image of PEA in mouse blood (**A**). The percentage of PEA in mouse whole blood (**B**). *P < 0.05, **P < 0.01 between the groups indicated

Platelets have been investigated whether they aggregate with eosinophils in the blood and BAL fluid of asthmatic patients, especially with asthma exacerbation [20]. Therefore, PEA formation was evaluated on the basis of cells containing CD41 and siglec-F in mouse whole blood using the FACS method. The percentage of PEA was increased in the positive control mice than in the negative control mice (19.4 % vs. 5.4 %). In contrast, the increased level of PEA (9.0 %) was significantly decreased by tirofiban treatment in mouse whole blood (*P < 0.05*) (Fig. 3). Moreover, the ratio of eosinophils which attached to platelets was calculated. These ratios were 1.26, 0.94, and 1.56 in control, OVA, and tirofiban groups, respectively. This result means that eosinophils attached to platelets are increased in asthmatic mice, and these cells are reduced after tirofiban treatment.

### Tirofiban inhibits both platelet and eosinophil activations in BAL fluid and lung tissue

**Fig. 4:**
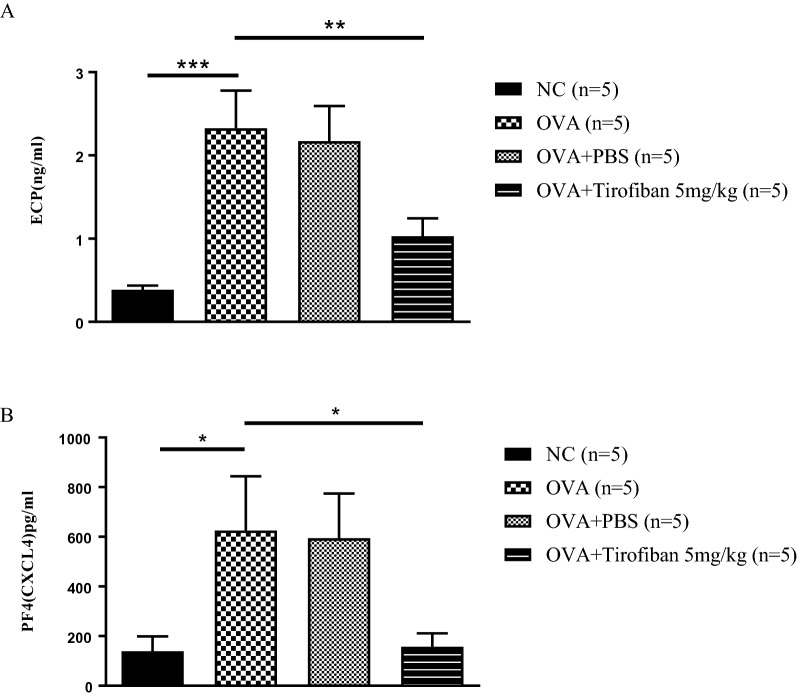
The activation of eosinophils and platelets in BAL fluid. The levels of ECP (**A**) and PF4 (**B**) in BAL fluid. *P < 0.05, **P < 0.01, ***P < 0.001 between the groups indicated

Next, the activation markers of platelets and eosinophils were measured in BAL fluid. The BAL fluid levels of PF4 and ECP were more elevated in the positive control mice than in the negative control mice. However, both activations markers were more effectively suppressed in the tirofiban-treated mice than in the positive control mice (*P < 0.05*) (Fig. 4).

**Fig. 5:**
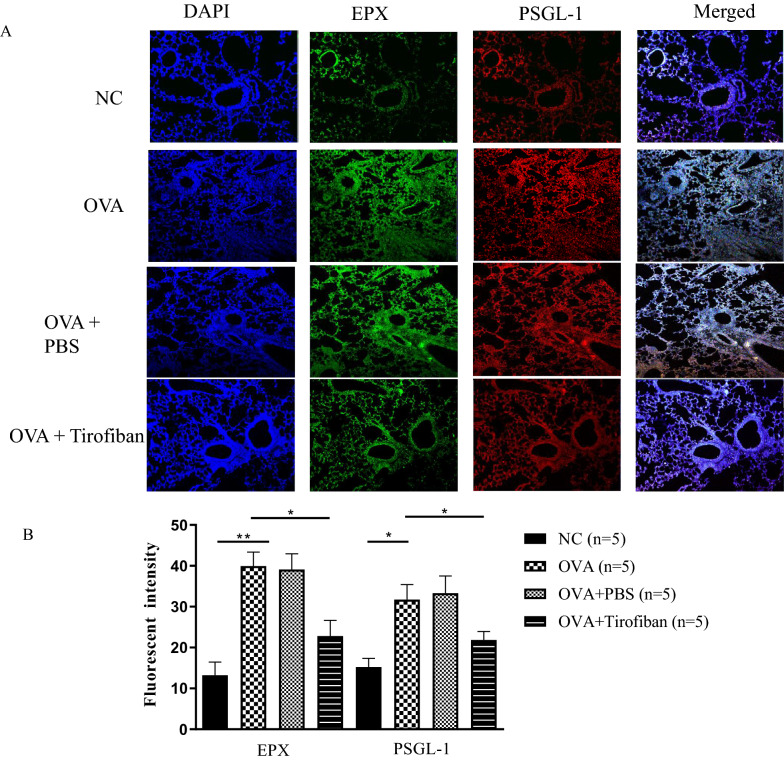
The levels of EPX and PSGL-1 in immunofluorescence staining (**A**), and the percentages of the quantified image (**B**). *P < 0.05, **P < 0.01 between the groups indicated

The activation markers were also evaluated in lung tissue. The levels of P-selectin and EPX were measured using the immunofluorescence method. The expression of EPX and PSGL-1 was markedly increased in the positive control mice, but the increased levels of EPX and PSGL-1 were significantly abrogated by tirofiban treatment in mouse lung tissue (*P < 0.05*) (Fig. 5A). The data are quantified in Fig. 5B.

### Tirofiban decreases adhesion molecule on eosinophil surface

**Fig. 6:**
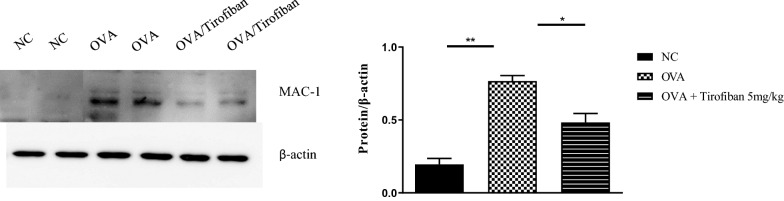
Detection of MAC-1 in lung homogenates. Western blot analysis of MAC-1 was performed in the lung of each group mice. *P < 0.05, **P < 0.01 between the groups indicated

MAC-1 on eosinophil is the well-known ligand of GPIIb/IIIa on platelet in PEA formation. Therefore, the levels of MAC-1 were measured in lung homogenate after tirofiban treatment by western blot. The expression levels of MAC-1 were significantly enhanced in the positive control mice compared to negative control mice, but these levels were significantly decreased by tirofiban treatment in lung homogenate (*P < 0.05*) (Fig. 6).

### The comparative effect of tirofiban and clopidogrel in the eosinophilic model of asthma

The GP IIb/IIIa receptor which can be blocked by tirofiban is a representative contact-dependent mediator for platelet–leukocyte interaction; in contrast, P2Y12 receptor which can be antagonized by clopidogrel is a soluble mediator related mediator on platelet–leukocyte interactions. Finally, their blocking effects were compared with our previous results in the eosinophilic asthma model [19]. When the results of OVA-positive control mice were designated as 100 %, the blocking effects of tirofiban and clopidogrel treatments were calculated as decreased percentages.

All asthma parameters, including AHR and type 2 inflammation markers, were decreased after clopidogrel or tirofiban treatments. However, the inhibitory effects of these 2 treatments were not significantly different.

## Discussion

Accumulating evidence suggests that platelets play a critical role in immunity and inflammation besides their well-known role as a cellular mediator in thrombosis. Platelets can contribute to the pathogenesis of inflammatory diseases such as atherosclerosis, transplant rejection, rheumatoid arthritis, and asthma directly through contact-dependent mechanisms or indirectly through secreted immune mediator-driven mechanisms [14]. Interactions between platelets and inflammatory cells usually promote pro-inflammatory action and can have a beneficial effect on the inflammatory process. However, exaggerated platelet interactions with leukocytes can lead to adverse effects of excessive immune stimulation and harmful inflammatory results. Increased platelet–neutrophil aggregation was found in acute coronary syndrome, which leads to cardiac dysfunction and vascular plaque formation [21]. Activated platelets aggregate with leukocytes in patients with inflammatory bowel disease [22], nephritis [23], or skin contact hypersensitivity [24]. The therapeutic effects of tirofiban have been suggested in some previous studies. Selective platelet inhibition by tirofiban resulted in reduced leukocyte–endothelial cell interactions in antigen induced arthritis [25], and antiplatelet therapy with tirofiban even showed effective improvement in the ventilation/perfusion ratio in COVID-19 patients with severe respiratory failure [26]. Clinical importance of platelet–leukocyte interactions, especially eosinophils, has been reported in asthmatic patients. Platelet depletion can reduce eosinophilic inflammation of the lung and decrease hyperresponsiveness in mouse asthma models [27]. Many eosinophils attached to platelets was observed in blood from asthmatic patients [8], and the administration of prasugrel, which is P2Y12 receptor antagonist showed decreased airway hyperresponsiveness compared to control treatment in a randomized, double-blind, placebo-controlled, cross-over study [28]. Taken together, the pathological role of platelets is strongly suggested in asthma, but not clearly understood especially as a therapeutic target.

Platelets can interact with other blood cells via 2 different ways: contact-dependent or soluble mediator-dependent. Our previous study demonstrated significant therapeutic effects of platelets and decreased PEA in a mouse asthma model after the blockade of P2Y12 which is a soluble mediator of platelets [19]. The GP IIb/IIIa receptor which is an integrin complex is one of the representative contact-dependent mediators of platelets to aggregate with other blood cells [29]. In this study, we evaluated the blocking effect of the GP IIb/IIIa receptor by tirofiban in an eosinophilic asthma model and found that AHR, eosinophilic inflammation, and the levels of type 2 inflammatory markers were significantly more decreased in the tirofiban-treated mice than in the positive control mice. To the best of our knowledge, this is the first report regarding the blocking effect of GP IIb/IIIa in asthma. So far, tirofiban has been used primarily in heart and vascular diseases [30, 31]. However, tirofiban has a therapeutic effect in chronic inflammatory diseases other than coronary disease by its involvement in the interaction between platelets and other blood cells [32]. Selective inhibition of the GP IIb/IIIa receptor by tirofiban significantly reduces platelet–endothelium interactions, which reduces knee-joint diameter in antigen-induced arthritis [25]. Although there are not a few publications about the blocking effect of GP IIb/IIIa in chronic inflammatory diseases other than coronary disease, other integrin families have been under investigation as therapeutic candidates due to their key role in cellular trafficking and activation [33, 34].

Importantly, PEA was more reduced in the tirofiban-treated mice than in the positive control mice and is the hallmark of allergic asthma after allergen challenge, which leads to platelet and eosinophil activations [27, 35]. Therefore, the activation levels of platelets and eosinophils were measured after the GP IIb/IIIa receptor was blocked with tirofiban. Platelets express a variety of chemokine receptors, immunoglobulin receptors, toll-like receptors, and adhesion molecules. They also store inflammatory mediators such as PF4 [36–39], and ECP is a well-known eosinophil chemoattractant and an augmenting agent for eosinophil adhesion [12]. We found that the activation of these markers was effectively suppressed by tirofiban treatment in BAL fluid and that the expression levels of EPX and PSGL-1 were significantly abrogated by tirofiban treatment in mouse lung tissue. PF4 and EPX are known to directly contribute to the induction of AHR in asthma models [40, 41]. Therefore, it is speculated that the therapeutic effects of tirofiban may rely on interactions between platelets and eosinophils.

Finally, we compared the blocking effects of tirofiban and clopidogrel administration in the same asthma model. As shown in Table 1, the therapeutic effect of clopidogrel seems more beneficial than that of tirofiban, albeit without any statistical significance. The P2Y12 receptor is more expressed in various immune cells than the GP IIb/IIIa receptor. Eosinophils, which are the most important effector cells in asthma, posses the P2Y12 receptor; dendritic cells, mast cells, lymphocytes, and vascular smooth muscle cells also express this receptor on their surface [42]. In contrast, the GP IIb/IIIa receptor is specifically expressed on platelets and have strong cell-specific properties. Therefore, the combination of clopidogrel and tirofiban would be warranted in future asthma studies.

**Table 1:** Comparisons between therapeutic effects of tirofiban and clopidogrel in asthma

	OVA (%)	Tirofiban 5 mg/kg (%)	Clopidogrel 10 mg/kg (%)	*P* value
Airway hyperresponsiveness	100	88.54	82.78	0.999
Total cell count in BAL	100	42.45	15.77	0.701
Eosinophil count in BAL	100	34.82	15.07	0.848
IL-4	100	49.85	35.56	0.885
IL-5	100	85.10	50.11	0.893
IL-13	100	38.21	10.40	0.657
PF4	100	39.08	18.65	0.562
ECP	100	44.29	24.90	0.736
Platelet-eosinophil aggregation	100	65.70	53.81	0.779

Data are expressed decreased as percentages when the results of OVA mice are designated as 100 %. *P* values were calculated for the comparisons between the tirofiban and clopidogrel groups

*AHR* airway hyperresponsiveness, *BAL* bronchoalveolar lavage, *PF4* platelet factor 4, *ECP* eosinophilic cationic protein

## Conclusions

The blocking of the GP IIb/IIIa receptor with tirofiban can significantly inhibit PEA as well as platelet and eosinophil activation, thereby attenuating AHR and airway inflammation in an eosinophilic asthma model. The results of this study suggest that the GP IIb/IIIa receptor can be used as a novel therapeutic target for asthma.

## Data Availability

All data and models used during the study are available from the corresponding author by request.

## Abbreviations

OVA: Ovalbumin
AHR: Airway hyperresponsiveness
GP: Glycoprotein
AERD: Aspirin-exacerbated respiratory disease
PEA: Platelet–leukocyte aggregation
PSGL-1: P-selectin glycoprotein ligand 1
ECP: Eosinophilic cationic protein
EPX: Eosinophil peroxidase
PF: Platelet factor
MCh: Acetyl-β-methylcholine chloride
BAL: Bronchoalveolar lavage
